# A Proteinaceous Fraction of Wheat Bran May Interfere in the Attachment of Enterotoxigenic *E. Coli* K88 (F4+) to Porcine Epithelial Cells

**DOI:** 10.1371/journal.pone.0104258

**Published:** 2014-08-13

**Authors:** Gemma González-Ortiz, Sílvia Bronsoms, H. C. Quarles Van Ufford, S. Bart A. Halkes, Ritva Virkola, Rob M. J. Liskamp, Cees J. Beukelman, Roland J. Pieters, José Francisco Pérez, Susana María Martín-Orúe

**Affiliations:** 1 Servei de Nutrició i Benestar Animal (SNiBA), Departament de Ciència Animal i dels Aliments, Universitat Autònoma de Barcelona, Barcelona, Spain; 2 Servei de Proteòmica i Biologia Estructural, Universitat Autònoma de Barcelona, Mòdul B Parc de Recerca, Barcelona, Spain; 3 Department of Medicinal Chemistry & Chemical Biology, Utrecht University, Utrecht, The Netherlands; 4 Department of Biosciences, General Microbiology, University of Helsinki, Helsinki, Finland; Cairo University, Egypt

## Abstract

Wheat bran (WB) from *Triticum aestivum* has many beneficial effects on human health. To the best of our knowledge, very little has been published about its ability to prevent pathogenic bacterial adhesion in the intestine. Here, a WB extract was fractionated using different strategies, and the obtained fractions were tested in different *in vitro* methodologies to evaluate their interference in the attachment of enterotoxigenic *Escherichia coli* (ETEC) K88 to intestinal porcine epithelial cells (IPEC-J2) with the aim of identifying the putative anti-adhesive molecules. It was found that a proteinaceous compound in the >300-kDa fraction mediates the recognition of ETEC K88 to IPEC-J2. Further fractionation of the >300-kDa sample by size-exclusion chromatography showed several proteins below 90 kDa, suggesting that the target protein belongs to a high-molecular-weight (MW) multi-component protein complex. The identification of some relevant excised bands was performed by mass spectrometry (MS) and mostly revealed the presence of various protease inhibitors (PIs) of low MW: Serpin-Z2B, Class II chitinase, endogenous alpha-amylase/subtilisin inhibitor and alpha-amylase/trypsin inhibitor CM3. Furthermore, an incubation of the WB extract with ETEC K88 allowed for the identification of a 7S storage protein globulin of wheat, Globulin 3 of 66 kDa, which may be one of the most firmly attached WB proteins to ETEC K88 cells. Further studies should be performed to gain an understanding of the molecular recognition of the blocking process that takes place. All gathered information can eventually pave the way for the development of novel anti-adhesion therapeutic agents to prevent bacterial pathogenesis.

## Introduction

The possibility that pathogens can be inhibited by naturally occurring anti-adhesive compounds is especially attractive and has captured significant attention [1]. Milk sources, plant-derived compounds, and microbial by-products have been the most important dietary products considered for this function [2]. Vegetable products with anti-adhesion activity are attractive candidates as therapeutic agents, because they are generally abundant. Although plant lectins are well represented in human and animal diets and many of these are well characterized [3], their application to anti-adhesion therapies is a recent strategy that has become an alternative to antibiotics. Theoretically, these lectins could interact with host-cell receptors to block adhesion by competition. But, additionally, they could interact with bacterial adhesins to enhance the clearance of bacteria by exclusion [4]. Numerous investigations involving plant extracts have recently been performed to reduce bacterial adhesion [5]–[7].

Wheat bran (WB) was first reported to modify intestinal microbiota by reducing the enterobacteria population in feces [8], [9]. Additional investigations showed a reduction of the *E. coli* population in the ileum digesta, and more interestingly, a reduction of the enterotoxigenic *E. coli* (ETEC) K88 attached to the ileum mucosa was observed when piglets received WB in their diet [10]. Even though the arabinoxylooligosaccharides of WB were initially considered to promote the anti-adhesive properties against enteropathogens [8], preliminary data suggested the involvement of a proteinaceous compound, which could specifically recognize ETEC K88 [11]. Therefore, the initial hypothesis of this work is that a protein or a glycoprotein present in the soluble extract of WB might interfere in the binding of ETEC K88 to the porcine epithelial cells, thus preventing the infectious process. Under this hypothesis, the aim of this study is to zoom in on the responsible proteinaceous compounds from the WB extract. To this end, a WB extract was fractionated using different strategies, and the obtained fractions were tested by different *in vitro* methodologies with the goal of identifying the putative candidates by mass spectrometry (MS).

## Material and Methods

### WB extraction and fractionation procedures

#### Obtaining the soluble extract of WB

The WB used in the study comes from a local Spanish mill (Moretó, Mollet del Vallès, Barcelona). First, the WB was finely ground in an analytical grinder and was then suspended in demineralized (DEMI) water to a solid-to-liquid ratio of 1∶10 (w/v). Subsequently, the suspension was sonicated (J.P. Selecta, S.A.) three times for 30 s each and then centrifuged (460×*g*, 5 min, 20°C). The supernatant extracted was divided into three aliquots. One aliquot was used just as it was to perform heat treatment and carbohydrate digestion. The second aliquot was freeze-dried and stored at room temperature (RT), and finally the third aliquot was immediately fractionated by molecular weight (MW).

#### Heat treatment

Wheat-bran extract was heated during 30 min at 90°C. The sample was cooled and stored at −20°C until use. The activity of the heated product was tested in the *in vitro* adhesion test.

#### Carbohydrate digestion

*O*-glycosidase (P0733S, 40,000,000 U/ml, New England BioLabs, Inc.) combined with Neuraminidase (N2876, 1,000 mU/ml, Sigma) was used to catalyze the hydrolysis of *O*-linked saccharides and *N*-acetyl-neuraminic acid from glycoproteins and oligosaccharides, maximizing the disappearance of sugars attached to proteins in the soluble extract of WB. The reaction was performed by mixing 2 mL of the soluble extract of 10% WB with 290 µL 10X G7 Reaction Buffer, 10 µL *O*-glycosidase (180 Units) and 25 µL Neuraminidase (11 Units). Samples were incubated at 37°C for 1.5 h with gentle shaking. A negative control was also included, which consisted of the same volume of the sample mixed with DEMI water, instead of the reaction buffer and enzymes. To stop the enzymatic reaction, the digested samples were heated at 60°C for 20 min. Samples were stored at −20°C.

#### Fractionation by MW

One aliquot of the soluble extract of WB was used to fractionate it by MW using Vivaspin 6 centrifugal concentrators (Sartorious) with a cut-off size of 300,000-Da. The upper part of the tube was filled with the soluble extract and centrifuged (3,000×*g*, 3.5 h, 4°C). After centrifugation, the upper part was adjusted with DEMI water to the same volume retrieved in the bottom container to achieve the same sample-volume. Two fractions were obtained: 1) >300-kDa and 2) <300-kDa. Finally, these fractions were immediately freeze-dried and kept at RT until further testing.

#### Size-exclusion chromatography (SEC)

To further fractionate the >300-kDa fraction, SEC was performed on an AKTA Purifier System (GE Healthcare) using a High Load Superdex 200 26/60 column (GE Healthcare). Thirty-five milligrams of the lyophilized >300-kDa fraction were injected into the column and the absorbance of the eluted fractions was monitored at 214 nm and 280 nm. Fractions were pooled in eight major fractions and were then freeze-dried. They were resuspended with DEMI water, at a final buffer concentration of phosphate buffered saline (PBS) 1X. The aliquots were stored at −20°C until use. In order to disrupt protein aggregations, the same separation was repeated adding acetonitrile (ACN) 20% to the chromatographic buffer. The fractions were pooled in six major fractions and after lyophilization, fractions were resuspended with DEMI water and kept at −20°C until their use.

### Efficacy validation tools

#### Bacterial strains

An enterotoxigenic *E. coli* (ETEC) K88 strain isolated from a colibacillosis outbreak in Spain [12] (Strain Reference n°: FV12408), serotype (O149:K91:H10 [K-88]/LT-I/STb), which was generously provided by the *E. coli* Reference Laboratory, Veterinary School of Santiago de Compostela University (Lugo), was used. ETEC K88 was cultured in unshaken Luria broth at 37°C [13]. Bacteria were serially cultured every 24 h, at least three times.

Bacterial cells were collected by centrifugation of 15 mL of an overnight culture (1,700×*g*, 5 min, 20°C). Supernatants were removed and PBS was added to the cell pellet to achieve an optical density (OD) of 1 (650 nm) for the bacterial suspension that was used in the adhesion test (approximately log 8.5–9 CFU [colony forming units]/mL). For the blocking test, the bacterial suspension was serially diluted to 6.5–7 log CFU/mL.

To isolate K88 fimbriae, wild-type ETEC strains FV12408 and 5/95 (O149, K88ac, LT+STb+; [14]) were cultured overnight at 37°C in tryptone soy broth (TSB, 100 mL) with mild shaking (50 rpm), or on Luria agar (tetracycline 12.5 µg/mL), respectively. Bacteria were collected and suspended in PBS. The bacterial suspensions were vortexed for two to four minutes to detach fimbrial filaments from bacterial surfaces [15]. Next, the bacteria were pelleted, and bacteria-free supernatants were analyzed in 15% SDS-PAGE gels. After Coomassie staining, the protein concentrations of fimbriae were determined densitometrically by the Tina (v2.0) image analysis program using bovine serum albumin (BSA, Sigma) as protein concentration standards in the gels.

#### Adhesion test (AT)

The ability of the different fractions to adhere to ETEC K88 was determined by using high-binding polystyrene microtitre plates in the *in vitro* AT as described by Becker et al. [16] and adapted by González-Ortiz et al. [11]. After different incubation and rinsing steps, the bacterial growth was monitored as OD at a wavelength of 650 nm at intervals of 10 min for 12 h (Spectramax 384 Plus, Molecular Devices Corporation). All readings were performed in two independent assays and in triplicate per trial. The OD data were translated to colony forming units (CFU) by using the linear models fitted by González-Ortiz et al. [11].

#### Blocking test (BT)

The cell-line characteristics, maintenance procedure and BT protocol were followed according to González-Ortiz et al. [17]. Wheat-bran extract and both fractions obtained by molecular weight (>300-kDa and <300-kDa) were subsequently submitted to a dose-response experiment. Each fraction was tested in triplicate in two independent assays. The OD data were translated to CFU by using the equations proposed by González-Ortiz et al. [17].

#### K88ac fimbrial binding to WB fractions

Binding of purified K88ac fimbriae to WB and the obtained fractions was tested in a dot-blot assay [18] using K88ac fimbriae purified from strain ETEC FV12408. Moreover, another purified K88ac fimbriae from ETEC strain 5/95, previously used for similar purposes [19], was used to compare the binding ability of these fimbriae extracts. To confirm K88 fimbrial-specific binding, we included casein glycomacropeptide (CGMP) and fetuin (Sigma) in the assay as positive control targets, as well as BSA, as a negative control [19]. All target proteins from the WB extract, >300-kDa, <300-kDa, CGMP, BSA (5 µg/dot), and chromatographic fractions (2.5 µl/dot), as well as fetuin (2.5 µg/dot), were immobilized on nitrocellulose membranes. After blocking for 1 h at 37°C in 2% (w/v) BSA/PBS, the membranes were washed three times with PBS containing 0.05% Tween 20 (PBS-Tween) and incubated with the purified K88 fimbriae (20 µg/ml in 1% BSA/PBS-Tween) overnight at 4°C with gentle shaking. Dot-blot membranes were washed three times with cold PBS-Tween and incubated with anti-FaeG polyclonal serum (diluted 1∶2000; [14]) for 2 h at 4°C. After washing, the membrane was incubated with alkaline phosphatase-conjugated anti-rabbit IgG (1∶1000; DakoCytomation) for 2 h at 4°C, and the bound proteins were visualized by bromochloroindolylphosphatenitrobluetetrazolium (Sigma).

#### Rescuing WB components that bind to fimbriae

To identify the possible molecular compounds of WB interacting with fimbriae, an incubation of the bacteria with the whole extract of WB was performed. For the experiment, we used the same bacterial conditions as described for AT. Enterotoxigenic *E. coli* K88 and WB extract (3∶1) were incubated for 30 min at 37°C; an incubation with PBS was also included as negative control. After the incubation, the bacteria cells were pelleted by centrifugation (1.700×*g*, 5 min, 20°C) and washed by hand pipetting with 1 mL of PBS. The washing step was repeated four times. Finally, a treatment with Triton X-100 at 1% for 10 min at RT eluted the WB compounds that interacted with the bacteria. The supernatant was filtered through 0.22-µm pore-size filters and kept at −20°C.

### Identifying the WB components that bind ETEC K88 fimbriae

#### One dimensional SDS-PAGE separation

Samples were treated with the 2D Clean-Up Kit (GE Healthcare) and were resuspended in lysis buffer (8 M urea, 2.5% Chaps, 2% ASB-14 and 40 mM DTT, pH 8.5). Samples were quantified using the Microplate BCA protein assay kit (Thermo Scientific). SDS-PAGE separation of fifteen microgram of each sample was performed using 12% acrylamide gels (BioRad), run at 15 mA for 30 min and 20 mA for 70 min. Proteins were stained with Instant Blue (Expedeon) for 1 h at RT.

#### Two dimensional SDS-PAGE separation

The proteins rescued after the incubation between ETEC K88 and WB were separated by 2D SDS PAGE. Samples were treated with the 2D Clean-Up Kit (GE Healthcare), resuspended in lysis buffer and quantified. 2D electrophoresis with immobilized pH gradients was carried out according to Bjellqvist et al. [20]. Briefly, 1D isoelectric focusing was performed on immobilized pH-gradient strips (7 cm, pH 3–10) using an Ettan IPGphor System. Samples (15 µg) were applied using cup-loading, and after focusing at 14 kVh, the strips were equilibrated for 15 min in an equilibration solution containing 10 mg/mL dithiothreitol (DTT) and then in an equilibration solution with 22.5 mg/mL iodoacetamide for 15 min on a rocking platform. 2D SDS-PAGE was performed by laying the strips on 12.5% precast gels (BioRad). The gels were run at RT at constant amperage (15 mA/gel) until the bromophenol-blue tracking front had run off the end of the gel.

#### Identification of proteins by MS

The selected protein spots were excised from gels and digested in-gel. Before tryptic digestion, samples were reduced by incubation with 10 mM DTT in 50 mM of ammonium bicarbonate for 30 min at RT, followed by alkylation with 25 mM iodoacetamide in 50 mM ammonium bicarbonate for 30 min at RT. Protein digestion was accomplished using 25 ng of trypsin sequencing grade (Promega) for 3 h at 37°C. Peptides were eluted by centrifugation with 50 µL of ACN:H_2_O (1∶1) +0.2% trifluoroacetic acid (TFA), evaporated using a speed-vacuum concentrator and resuspended in 5 µL of H_2_O+0.1% TFA. For the MS analysis, all samples were prepared by mixing 0.5 µL of sample with the same volume of a solution of α-cyano-4-hydroxycinnamic acid matrix (10 mg/mL in 30% ACN, 60% water +0.1% TFA) and were spotted onto a ground-steel plate (Bruker Daltonics) and allowed to air-dry at RT. Matrix-assisted laser desorption/ionization time-of-flight (MALDI-TOF) mass spectra were recorded in the positive ion mode on an Ultraflextreme mass spectrometer (Bruker Daltonics). Ion acceleration was set to 25 kV. All mass spectra were externally calibrated using a standard peptide mixture. For peptide-mass fingerprint analysis, the Mascot search engine (Matrix Science) was used with the following parameters: NCBInr database, 2 maximum-missed trypsin cleavages, cysteine carbamidomethylation and methionine oxidation as variable modifications and 50 ppm tolerance. Positive identifications were accepted with scores over the significance threshold and P<0.05.

### Statistical analysis

The statistical analyses were performed using SAS 9.2 [21]. The OD data from the AT and the BT were processed by non-linear regression analysis using the non-linear P-NLIN (Gauss-Newton method) procedure following the equations described by Becker et al. [16]. From the time at which the bacterial growth reached an OD of 0.05 (t_OD = 0.05_, h), the log CFU were calculated for each fraction using the described linear models for each *in vitro* test [11], [17]. Significant differences in the log CFU among fractions were determined by one-way analysis of variance (ANOVA), by using the GLM procedure. Linear, quadratic and cubic contrasts were performed to analyze the dose-response of WB and the >300-kDa fraction in the BT. Differences between means were tested by the Tukey-Kramer adjustment for multiple comparisons. Differences with P values <0.05 were considered to be statistically significant.

## Results and Discussion

### Nature of the putative compound of WB involved in ETEC K88 adhesion

Since anti-adhesives based on carbohydrate structures are more common than are those based on proteins [22], [23], a carbohydrate digestion was carried out by using both *O*-glycosidase and neuraminidase in order to detect carbohydrate participation in the ETEC K88 binding. The *O*-glycosidase, also known as endo-α-N-acetylgalactosaminidase, catalyzes the removal of Core 1 (Galβ(1-3)GalNAc-α-O-Ser/Thr) and Core 3 (GlcNAcβ(1-3)GalNAc-α-O-Ser/Thr) *O*-linked disaccharides from glycoproteins. On the other hand, the neuraminidase is an exoglycosidase enzyme which catalyzes the hydrolysis of α2-3-, α2-6-, and α2-8- linked *N*-acetyl-neuraminic acid residues from glycoproteins and oligosaccharides, thus maximizing the disappearance of sugars attached to proteins. The digestion of the soluble extract of WB did not modify its adhesive ability, as compared to the non-incubated WB (data not shown), indicating that these carbohydrates do not play any important role in the recognition of ETEC K88.

On the other hand, the heat treatment at 90°C for 30 min reduced the number of ETEC K88 attached to wells to levels similar to those of PBS, as compared to the WB extract, which significantly attached more bacteria (5.42 vs. 5.39 vs. 6.68 log CFU, per well, respectively; *P*<0.0001). This result is consistent with the involvement of a proteinaceous compound from WB in the attachment of ETEC K88. In this sense, some reports have suggested the ability of protein fractions from plants to act as anti-adhesive substrates [22], [24]. Wheat bran is a by-product with a protein content of about 151 g/kg to 221 g/kg dry matter [25]. Nonetheless, other components in the WB could also interfere with the binding of ETEC K88 to epithelial cells, such as phytic acid (42 mg/g), polyphenols (3.20 mg/g), tannins (2.9 mg/g), saponins (2.7 mg/g) and trypsin inhibitors (54.2 U/g) [26]. For example, the proanthocyanidins of cranberry extracts are known to prevent urinary tract infections by disrupting the binding between the bacteria and the uroepithelial receptors and by changing the physicochemical surface properties of *E. coli* [27]. However, most of them are non-protein in nature and heat-resistant. Thus, they could be discarded in the current identification of the active compound recognizing ETEC K88 due to the loss of activity when the WB-extract was heated.

### Identification of the MW fraction that contributes in the recognition of ETEC K88

**Figure 1:**
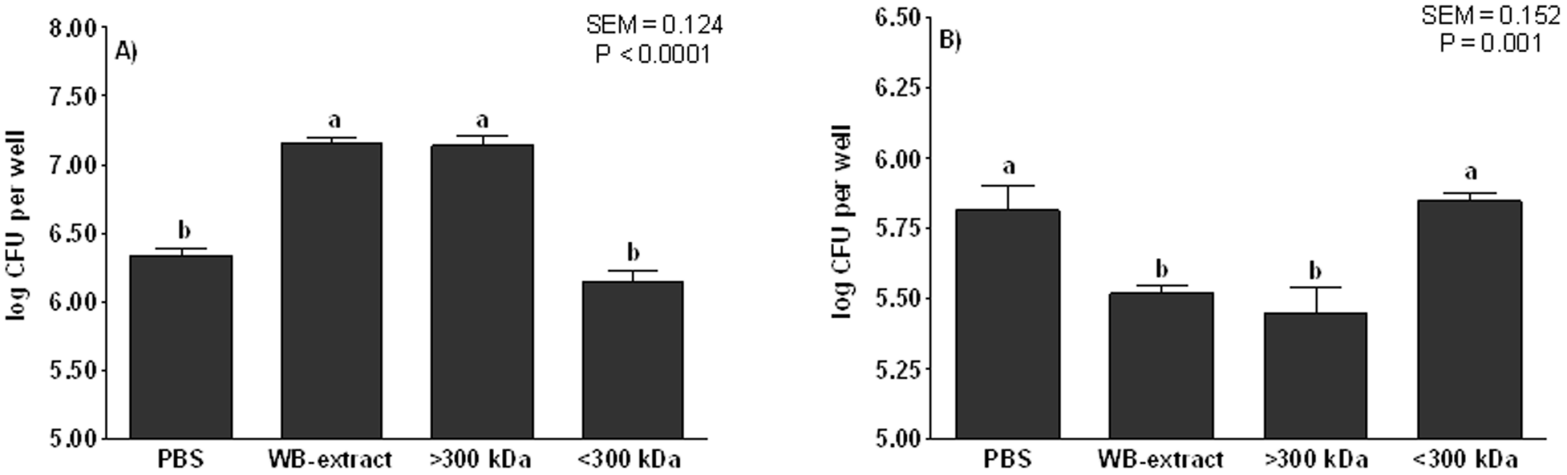
The adhesive (Figure A) and anti-adhesive (Figure B) abilities of fractions evaluated using the enterotoxigenic *E. coli* (ETEC) K88. A) Number of bacteria (log CFU per well) attached to wells coated with the different molecular-weight (MW) fractions obtained from wheat bran (WB) in the *in vitro* adhesion test (AT). The higher the log CFU counts than those of PBS, the higher the adhesive ability. The samples tested were the WB extract (14 mg/ml), the >300-kDa fraction (2.7 mg/ml) and the <300-kDa fraction (17 mg/ml). B) Number of bacteria (log CFU per well) that attached to IPEC-J2 cells after being co-incubated for 30 min with the different fractions obtained from WB. The lower the log CFU counts than those of PBS, the higher the blocking-adhesion ability. Different letters mean significant differences (P<0.05) between fractions. Data result from the experiments performed in triplicate in two independent assays. Error bars represent the standard error of the mean.

Fractionation by MW of the soluble extract of WB using a 300,000-Da cut-off- size filter resulted in two different fractions: >300-kDa and <300-kDa. The sample concentrations included in the *in vitro* AT and BT were fixed according to an equivalent of an extract at 10%. Therefore, the WB extract and the >300-kDa and <300-kDa fractions were tested at 14 mg/mL, 2.7 mg/mL and 17 mg/mL, respectively. Results obtained in the AT as well as in the BT with the different fractions (Figure 1) revealed that the fraction adhering to and blocking ETEC K88 attachment to IPEC-J2 was the >300-kDa; whereas the <300-kDa fraction did not modify the number of ETEC K88 adhered to or blocked, when compared to PBS in both *in vitro* assays.

**Figure 2:**
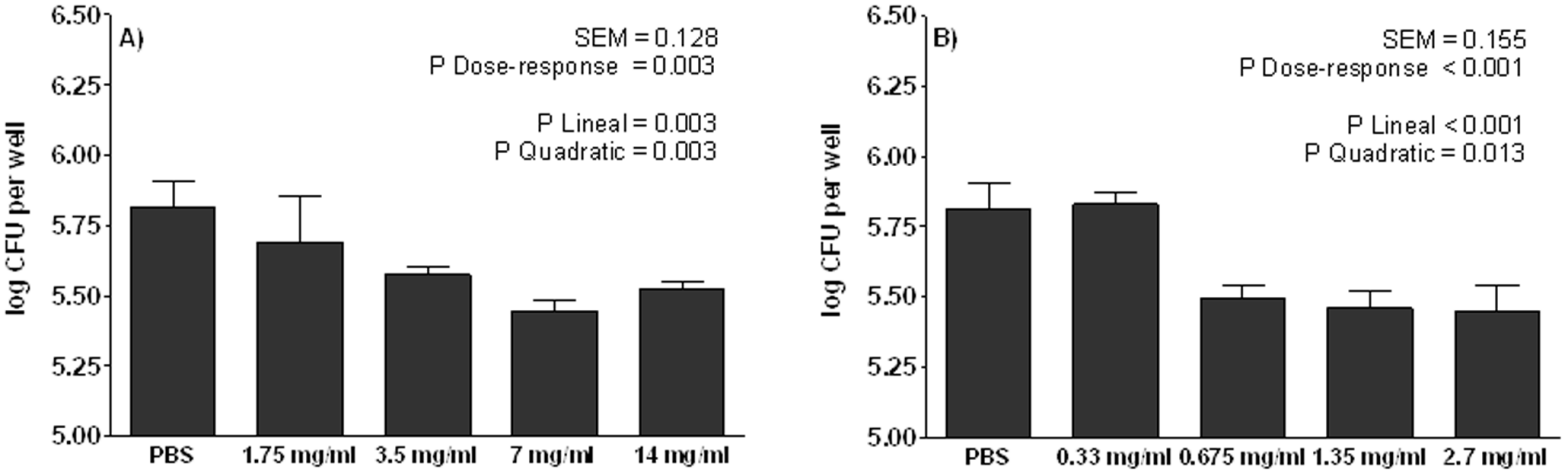
Dose-response of WB extract (A) and the >300-kDa fraction (B) to block the attachment of ETEC K88 to IPEC-J2. Log CFU: number of bacteria attached to the intestinal cells that were not blocked, when compared to those of PBS. The lower the log CFU counts, the higher the blocking-adhesion ability. Linear, quadratic and cubic contrasts were performed to analyze the dose-response. Data result from the experiments performed in triplicate in two independent assays. Error bars represent the standard error of the mean.

A dose-response assay was performed with the WB extract and the >300-kDa fraction in a wide range of concentrations (Figure 2). The linear and quadratic responses were significant for different doses in the WB extract (P<0.05) (Figure 2A) and also in the >300-kDa fraction (Figure 2B), demonstrating their anti-adhesive ability.

On the basis of these results, a proteinaceous compound from the >300-kDa fraction seems to mediate the recognition of ETEC K88. Therefore, a 1D SDS-PAGE was performed to compare the protein profile among the WB extract and the >300-kDa and the <300-kDa fractions to detect a specific band that could be involved in the fimbriae recognition process. Different protein profiles were visualized in the 1D SDS-PAGE (data not shown). However, almost no bands in the <300-kDa fractions were detected. The WB extract and the >300-kDa fraction shared several protein bands, and many of them displayed a MW below 90 kDa. This fact is quite unexpected, since one of the fractions should mostly contain proteins with a MW higher than 300-kDa. These findings could indicate that the target protein belongs to a high-MW multicomponent-protein complex (>300-kDa) which is disrupted under the denaturing conditions of the SDS gel and renders the individual proteins. Also the possibility that the molecules of the sample could not be fully separated by their MW using the centrifugal concentrators should not be discarded.

### Further characterization of the high-MW fraction by SEC

**Figure 3:**
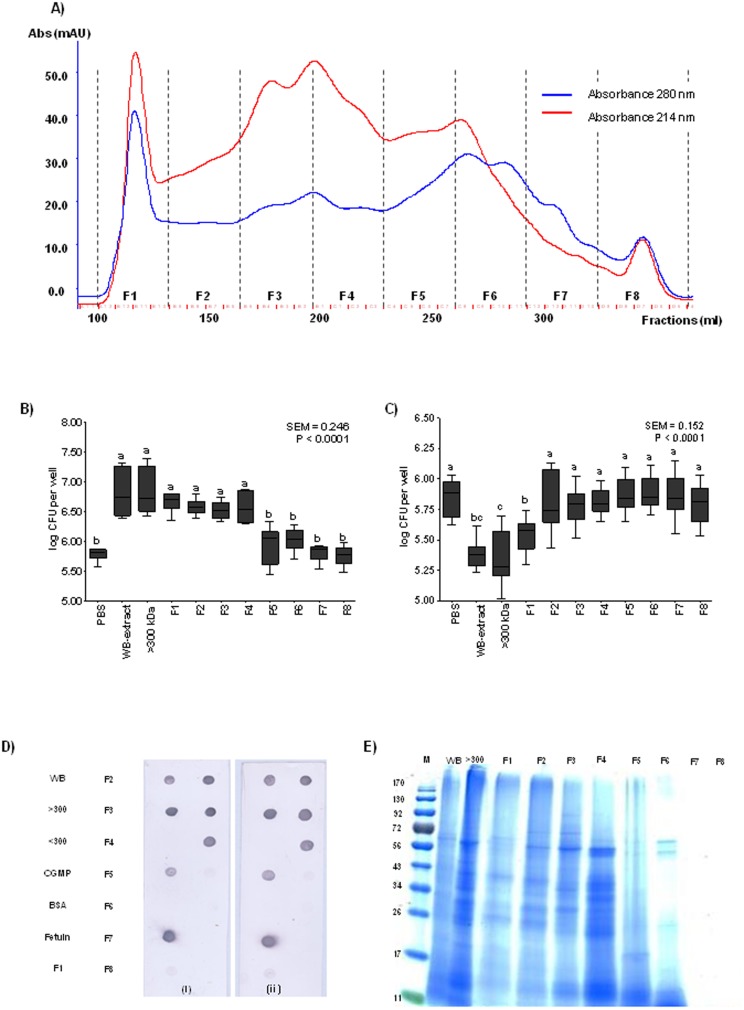
Recognition of ETEC K88 to the size-exclusion chromatography (SEC) fractions obtained. A) SEC of the >300-kDa fraction. Distribution of the eight fractions obtained. B) Number of bacteria (log CFU per well) attached to wells coated with the different fractions in the *in vitro* adhesion test (AT). The higher the log CFU counts than those of PBS, the higher the adhesive ability. C) Number of bacteria (log CFU per well) that attached to IPEC-J2 cells in the blocking test (BT). The lower the log CFU counts than those of PBS, the higher the blocking-adhesion ability. In Figures B and C, different letters mean significant differences (P<0.05) between fractions. Data result from two independent assays performed in triplicate. Error bars represent the standard error of the mean. D) Dot-blot analysis with purified fimbriae on immobilized WB extract, >300-kDa and <300-kDa fractions, CGMP, BSA, fetuin and the eight fractions (F1 to F8). (i) Binding of purified K88ac fimbriae of ETEC strain FV12408. (ii) Binding of purified K88ac fimbriae of ETEC strain 5/95. E) One-dimension SDS-PAGE of the eight fractions obtained by SEC.

**Figure 4:**
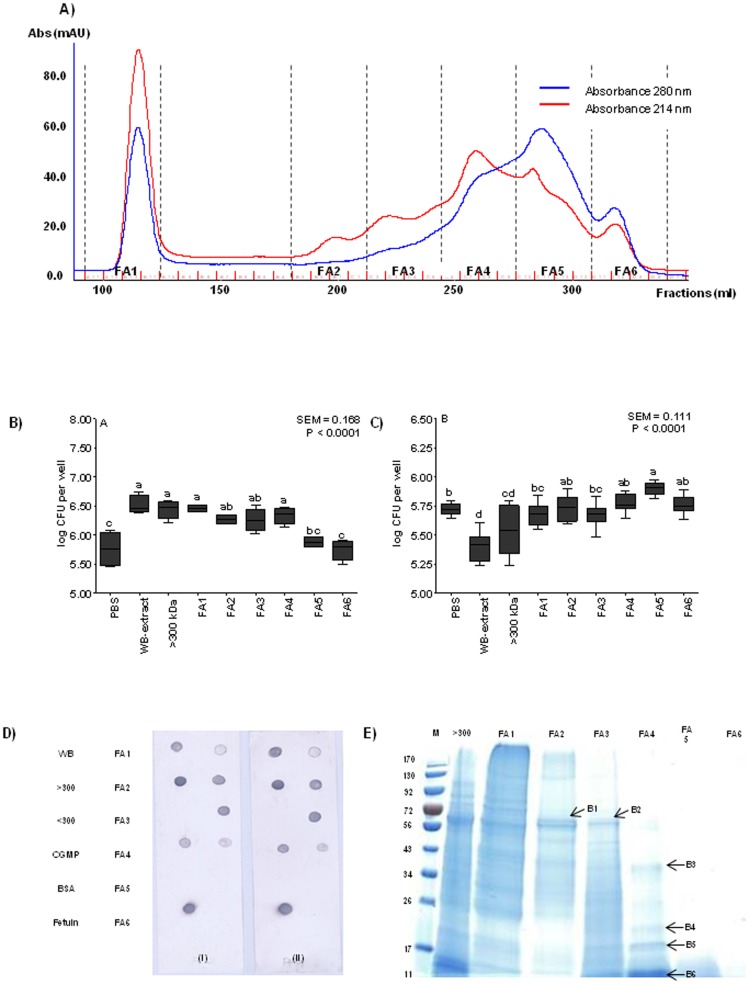
Recognition of ETEC K88 to the size-exclusion chromatography (SEC) fractions obtained with acetonitrile (ACN). A) SEC of the >300-kDa fraction treated with solvent. Representation of the six fractions obtained. C) Number of bacteria (log CFU per well) attached to wells coated with the different fractions in the *in vitro* adhesion test (AT). The higher the log CFU counts than those of PBS, the higher adhesive ability. D) Number of bacteria (log CFU per well) that attached to IPEC-J2 cells in the blocking test (BT). The lower the log CFU counts than those of PBS, the higher the blocking-adhesion ability. In Figures C and D, different letters mean significant differences (P<0.05) between fractions. Data result from two independent assays performed in triplicate. Error bars represent the standard error of the mean. D) Dot-blot analysis with purified fimbriae on immobilized WB extract, >300-kDa, <300-kDa, CGMP, BSA, fetuin and the six fractions (FA1 to FA6). (i) Binding of purified K88ac fimbriae of ETEC strain FV12408. (ii) Binding of purified K88ac fimbriae of ETEC strain 5/95. E) One-dimension SDS-PAGE of the six fractions obtained by SEC treated with solvent. From B1 to B6 are indicated the excised bands which were identified by MS.

Size-exclusion chromatography (SEC) was performed to fractionate the >300-kDa fraction and to isolate the target protein. Eight SEC fractions (Figure 3A) were obtained (F1 to F8) and subsequently evaluated in the in *vitro* AT and BT and dot-blot assay. Results obtained from the AT revealed that F1, F2, F3 and F4 had roughly the same number of ETEC K88 cells bound as displayed by the >300-kDa fraction and the WB extract (Figure 3B). Fractions from F5 to F8 were not able to bind ETEC K88, as was expected due to the low-MW proteins that were contained in these samples. When the same fractions were evaluated in the BT assay, the main difference was found in the F1 fraction, which was the only one able to interfere in the ETEC K88 adhesion to IPEC-J2 by reducing the number of attached bacteria (Figure 3C). This result shows that the chromatographic separation allowed us to isolate the protein with blocking activity of the >300-kDa sample in a single Fraction, F1. Also, it indicates that there are two distinct functions in the >300-kDa sample, an adhesive property observed in Fractions F1 to F4, and a blocking function located in Fraction F1 which may works simultaneously as a receptor analog and as an adhesin analog [3]. In Figure 3D, the results of the dot-blot assay are represented. Both K88ac fimbriae, coming from two different enterotoxigenic *E. coli* strains (FV12408 and 5/95), bind to the WB extract and >300-kDa fraction but not to the <300-kDa fraction. Regarding the eight SEC fractions obtained from the >300-kDa fraction, K88ac fimbriae bind strongly to F2, F3 and F4 fractions, but less intensely to F1. Similarly to the AT and BT assays, no binding signals were detected for F5, F6, F7 and F8 fractions. The eight SEC fractions were further separated by SDS-PAGE gel and compared (Figure 3E). Surprisingly, F1, F2, F3 and F4 contain several low-MW proteins. The presence of these low-MW proteins in fractions belonging to the high-MW region of the SEC and coming from a sample previously separated by a filter of 300-kDa might be due to the existence of protein complexes in the >300-kDa fraction and the SEC, which are individualized under the denaturing conditions of the gel. Accordingly, the >300-kDa fraction was treated with ACN and subsequently fractionated by SEC in the presence of ACN to break down the potential complexes without completely denaturing the proteins, in order to keep their activity. The obtained chromatographic profile differed from the previous SEC, where it could be observed that fractions F2 and F3 had nearly disappeared, which seems to indicate that the ACN treatment had an effect on the sample separation reinforcing the idea of a multi-component complex. As a result of this chromatography (Figure 4A), six fractions were obtained (FA1 to FA6) and evaluated by both *in vitro* AT and BT and the dot blot assay. In the AT, SEC fractions FA1 to FA4 showed a similar effect to that of the >300-kDa fraction and WB extract, binding a high log CFU of ETEC K88 (Figure 4B) and showing that the ACN treatment did not affect their binding capacity. These results are in accordance with the dot-blot results (Figure 4D), which show that both types of K88ac fimbriae bind strongly to FA2 and FA3, but with lower intensity to FA1 and FA4. In contrast, those active fractions in the AT and the dot-blot assay were not able to interfere in the ETEC K88 attachment to IPEC-J2 (Figure 4C). These results seem to indicate that the interference of the attachment to IPEC-J2 would be due to a protein complex, while the adhesive activity might be mediated by individual proteins. The 1D SDS-PAGE electrophoresis did not reveal a clear, different band pattern among fractions (Figure 4E).

In the light of these results, six bands (B1, B2, B3, B4, B5 and B6) which were in common among the active fractions in the AT and the dot-blot assay (FA2 to FA4) were excised to identify the proteins by MALDI-TOF MS. Table 1 summarizes the most important information regarding the identification of the excised bands by MS using the Mascot search engine. Bands B1 and B2 shared the same beta amylase and B1 also contained a protein disulphide isomerase 2 precursor of 56-kDa. In FA4, the high intensity of bands distributed at lower MW allowed us to excise more bands for identification. Serpin-Z2B and Class II chitinase were found to be the most representative proteins in the B3 and B4 bands, respectively. Finally, two protease inhibitors were identified in B5 and B6 with a low-MW of approximately 19-kDa.

**Table 1:** Identification of proteins present in the soluble extract of wheat bran (*Triticum aestivum*) (WB) after fractionation by 1D SDS gel (Spot numbers from B1 to B6) and identification of proteins rescued after incubation between ETEC K88 cells and WB extract (Spot numbers from B7 to B9).

Spot No.	Accession number	Protein description	Taxonomy	Mascot Score	Protein MW (Da)	Pep No.^a^	Seq. Cov. (%)^b^
B1	gi|474451266	Beta amylase	*Triticum urartu*	93	58,710	14	29
	gi|13925726	Protein disulfide isomerase 2 precursor	*Triticum aestivum*	70	56,406	8	24
B2	gi|474451266	Beta amylase	*Triticum urartu*	131	58,710	14	28
B3	gi|473793747	Serpin-Z2B	*Trititicum urartu*	79	45,112	12	36
B4	gi|62465514	Class II chitinase	*Triticum aestivum*	64	28,200	13	87
B5	gi|123975	Endogenous alpha-amylase/subtilisin inhibitor	*Triticum aestivum*	313	19,621	21	92
B6	gi|123957	Alpha-amylase/trypsin inhibitor CM3	*Triticum aestivum*	85	18,209	6	57
**Proteins rescued after incubation between ETEC K88 and WB-extract**							
B7	gi|260870699	RNA polymerase, beta subunit	*Escherichia coli*	286	155,048	32	26
B8	gi|215398470	Globulin 3	*Triticum aestivum*	122	66,310	21	41
B9	gi|300946929	Translation elongation factor Tu	*Escherichia coli*	342	44,822	41	94

a Number of matched peptides.

b Sequence coverage of the matched peptides in percentage.

We have therefore identified, among the active chromatographic fractions, different candidate proteins from WB that could be involved in its anti-adhesives properties. From these candidate proteins, or their families, it has been possible to find different reports demonstrating anti-microbial properties [28]–[35], supporting them as putative candidates. Our study does not give full evidence of their implication on the anti-adhesive properties, but more studies will be needed to evaluate their possible implication.

### Isolation of wheat-bran proteins attaching to ETEC K88

**Figure 5:**
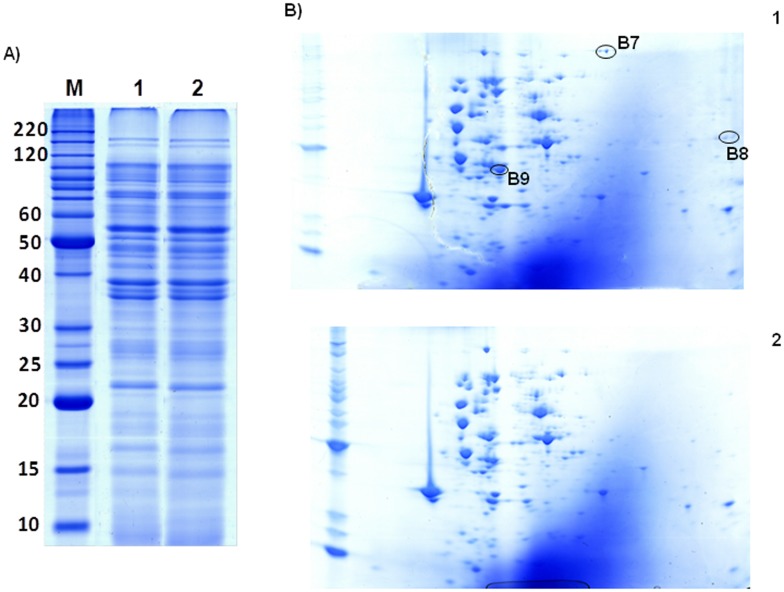
Isolation of wheat-bran proteins attaching to ETEC K88. ETEC K88 cells were incubated with wheat-bran extract (1) and PBS (2). The proteins obtained after the shaving process were separated in 1D (A) and 2D (B) gels. The spots with a fold >2 are labeled as B7, B8 and B9.

In parallel, an *in vitro* experiment was conducted which consisted of incubating ETEC K88 cells with the WB extract in order to rescue the WB molecules most firmly attached to the fimbriae. This procedure has been called “surfomics” by some authors and allows for the identification of surface proteins through the shaving of the exposed proteins of living cells [36]. The material retrieved after the shaving process was used to perform 1D and 2D gels with both samples and to identify the spots which were only detected in the presence of WB (Figure 5). In the 1D gel it was not possible to identify any differential bands between the incubation of bacteria with PBS or WB extract due to the high number of proteins in the sample. The two 2D gels were compared using the Progenesis Same Spots software (Nonlinear Dynamics) and, after the alignment of the images, six spots with a fold >2 were detected. Three of the spots belonged to the same train of spots (Figure 5b, Spot Number B7) and contained a bacterial protein (See Table 1), as did Spot Number B9. Finally, two spots belonging to the same train of spots (Figure 5b, Spot Number B8) were identified as a protein from *Triticum aestivum*, Globulin 3 (See Table 1). Up to now, few studies have sought to characterize 7S wheat globulins because they were thought to be minor storage proteins. However, Teodorowicz et al. [37], [38] have recently demonstrated the modulation of human-gut microbiota proliferation, survival and adhesion by 7S peanut globulin, even it has been reported anti-bacterial activity for soy globulins [39].

## Conclusions

Taken together, our results suggest that the anti-adhesive properties of the WB extract may respond to different mechanisms. It is hypothesized that a high-molecular-weight complex above 300 kDa is involved in blocking ETEC K88 attachment to IPEC-J2. The identification of several proteins with adhesive properties reinforces the belief that they would need to be joined together in a more complex structure to fully interfere in the adhesion of ETEC K88 to pig intestine. In this work different candidate proteins are proposed that could be involved in these antimicrobial properties. However, further studies should be performed to validate the results and gain an understanding of the recognition and blocking processes that take place. All of the gathered information would eventually pave the way for the design of novel therapeutic agents to prevent bacterial pathogenesis.
